# Supplementary material for: Chicks change their pecking behaviour towards stationary and mobile food sources over the first 12 weeks of life: improvement and discontinuities
Source: PeerJ. 2014 Oct 23;2:e626. doi: 10.7717/peerj.626 (PMC4217182; doi:10.7717/peerj.626)
Supplement: Table S1 [file peerj-02-626-s002.pdf]

Table 2. Mean within bout, peck rate after 30 seconds of food crumb pecking, n= 30 chicks

Age (days)	11	29	41	53	65
(wks)	2	5	6	8	10
Peck Rate (pecks/min)	151.16	170.01	201.37	178.99	166.30
S.D.	46.25	43.99	38.58	45.76	30.78
N	30	30	30	30	30
